# Synaptic Plasticity of Human Umbilical Cord Mesenchymal Stem Cell Differentiating into Neuron-like Cells In Vitro Induced by Edaravone

**DOI:** 10.1155/2018/5304279

**Published:** 2018-10-28

**Authors:** Yunpeng Shi, Chengrui Nan, Zhongjie Yan, Liqiang Liu, Jingjing Zhou, Zongmao Zhao, Depei Li

**Affiliations:** ^1^Department of Neurosurgery, The Second Hospital of Hebei Medical University, Shijiazhuang, Hebei 050000, China; ^2^Neuroscience Research Center, Hebei Medical University, Shijiazhuang, Hebei 050000, China; ^3^Department of Anesthesiology and Critical Care, The University of Texas MD Anderson Cancer Center, Houston, TX 77030, USA

## Abstract

**Objective:**

The human umbilical cord mesenchymal stem cells (hUMSCs) are characterized with the potential ability to differentiate to several types of cells. Edaravone has been demonstrated to prevent the hUMSCs from the oxidative damage, especially its ability in antioxidative stress. We hypothesized that Edaravone induces the hUMSCs into the neuron-like cells.

**Methods:**

The hUMSCs were obtained from the human umbilical cord tissue. The differentiation of hUMSCs was induced by Edaravone with three different doses: 0.65 mg/ml, 1.31 mg/ml, and 2.62 mg/ml. Flow cytometry was used to detect the cell markers. Protein and mRNA levels of nestin, neuron-specific enolase (NSE), and glial fibrillary acidic protein (GFAP) were detected by Western blot and RT-PCR. The expression of synaptophysin (SYN), growth-associated protein 43 (GAP43), and postsynaptic density 95 (PSD95) was detected by Real-Time PCR.

**Results:**

As long as the prolongation of the culture, the hUMSCs displayed with the long strips or long fusiform to fat and then characterized with the radial helix growth. By using flow cytometry, the cultured hUMSCs at the 3rd, 5th, and 10th passages were expressed with CD73, CD90, and CD105 but not CD11b, CD19, CD34, CD45, and HLA-DR. Most of the hUMSCs cultured with Edaravone exhibited typical nerve-immediately characters including the cell body contraction, increased refraction, and protruding one or more elongated protrusions, which were not found in the control group without addition of Edaravone. NSE, nestin, and GFAP were positive in these neuron-like cells. Edaravone dose-dependently increased expression levels of NSE, nestin, and GFAP. After replacement of maintenance fluid, neuron-like cells continued to be cultured for five days. These neuron-like cells were positive for SYN, PSD95, and GAP43.

**Conclusion:**

Edaravone can dose-dependently induce hUMSCs to differentiate into neuron-like cells that expressed the neuronal markers including NSE, nestin, and GFAP and synaptic makers such as SYN, PSD95, and GAP43.

## 1. Introduction

Stem cells are characterized with self-renewal ability and multidirectional differentiation potential. For example, bone marrow, umbilical cord, and epidermis [1–3] were able to differentiate into various functional cells induced by different approaches [4]. In particular, mesenchymal stem cells (MSCs), as one of pluripotent stem cells, have been demonstrated to be capable to differentiate into pluripotent cells [5] including vascular endothelial cells [6], neuron-like cells [7, 8], corneal endothelial cell [9], and hepatocyte-like cells [10]. The differentiation ability probably explains why MSCs have been reported to play important roles in neuronal protection from the oxidative stress, inhibition of ischemia-induced necrosis, and apoptosis [11]. These evidences indicate the probability that the induction of MSC differentiation for neuronal protection would be an effective method for eliminating brain ischemia injury in a clinical setting. The human umbilical cord MSCs (hUMSCs) have the advantages of simple convenient preparation, feasible source, nontraumatic risk of infection, and its low immunogenicity and immunosuppression, so the hUMSCs turn to be an ideal source used as the engineering cells in studying stem cell differentiation.

Edaravone, a low-molecular weight agent, can scavenge oxygen free radicals and decrease the ability of the xanthine oxidase and hypoxanthine oxidase and reduce the formation of prostacyclin, thus enhance the tissue antioxidative capacity [12]. Edaravone can penetrate the blood-brain barrier, and it has been used in clinic to decrease the ischemia-induced injury in the brain such as acute cerebral infarction, cerebral hemorrhage, and even amyotrophic lateral sclerosis [13]. The early treatment of acute cerebral infarction with Edaravone can prevent the reduction of cerebral blood flow around the lesion area and increase the neuronal antioxidant ability. More importantly, Edaravone was reported to prevent the MSC damage from hypoxia and activate the potential for angiogenesis [14], but it is not known whether Edaravone can induce the differentiation of hUMSCs into the neuronal-like cells that would be explained as another mechanisms underlying its benefits to treat ischemia-induced neuronal injury. The aim of this study was to observe the effects of Edaravone on the differentiation of hUMSCs into neuron-like cells and to further explore the possible mechanisms.

As the main part of the neuron transmission of information, synapses are the structural basis of the interconnected transmission of information between neurons. It is the basic structure and functional unit of the neural loop. The establishment and maintenance of synapses depend on the corresponding expression of many genes. The number and density of synaptophysin (SYN) can indirectly reflect the number and density of synapses [15]. GAP43 (growth-associated protein 43) is closely related to axonal growth and is a key factor for axonal growth and elongation [16]. PSD95 (Postsynaptic density protein 95) is the most important scaffold protein on the postsynaptic membrane, which plays an important role in the process of synapse formation. We use Edaravone to induce hUMSCs to differentiate into nerve-like cells. We continue to culture the cells and detect the expression of specific synapse markers, SYN, GAP43, and PSD95, which lays the foundation for further cell electrophysiological study.

## 2. Materials and Methods

### 2.1. Materials and Chemicals

The umbilical cord was obtained from the Department of Obstetrics and Gynecology of the Second Affiliated Hospital of Hebei Medical University. All the participants signed the relevant consent letter to use the umbilical cord for the present research. The following reagents were obtained commercially: Edaravone (packing: 20 g, purity: 99.7%, batch no.: A06-131204) was provided by Jilin Province Boda Pharmaceutical Co. Ltd. FBS (BI, Israel); L-DMEM/F-12, H-DMEM/F-12 (GIBCO, Grand Island, USA); EDTA and DMSO (Sigma-Aldrich Co. LLC., St. Louis, MO 63178,USA); FITC-CD19, FITC-CD34, PE-CD11b, PE-CD73, PE-CD90, PE-CD45, and PE-CD105 (Becton, Dickinson and Company, Franklin Lakes, New Jersey 07417-1880); PS immunohistochemistry kit (Beijing Zhongshan Golden Bridge Company, China); Taq PCR star mix (Genstar, China); EasyScript First-Strand cDNA synthesis supermix (TransGen Biotech, China); TB Green™ Premix Ex Taq™ II (Tli RNaseH Plus) (TaKaRa Japan); cell *RIPA* Lysis Buffer and phenylmethylsulfonyl fluoride (Solarbio, Beijing, China); polyclonal antibodies of neuron-specific enolase (NSE) and nestin (OriGene, USA); glial fibrillary acidic protein (GFAP) and glyceraldehyde-3-phosphate dehydrogenase (Affbiotech, USA); and polyclonal antibodies of synaptophysin, growth-associated protein 43, and postsynaptic density 95 (Abways, China).

### 2.2. Isolation and Culture of hUMSCs

The obtained umbilical cords were placed in H-DMEM/F12 culture medium under aseptic conditions, stored at 4°C, and then timely transported to a cell culture room to carry out the following steps. Each umbilical cord was rinsed thoroughly with D-Hank's medium. After removing the blood sample, umbilical artery, and umbilical vein, the umbilical cord mesenchymal tissue was cut into pieces of 1 mm^3^ in size, digested with 0.2% collagenase II, and placed in a culture flask containing 2 ng/ml EGF, 20% FBS, 25 mM L-Glu, and 100 U/ml penicillin-streptomycin mixture at 37°C with 5% CO_2_ and saturation humidity to obtain primary cells. Half of the culture medium was replaced every 24 h, and it was replenished every 3 days. When the cells achieved 80–90% confluency, the medium was removed, and cells were rinsed two times with PBS and then digested with trypsin (0.25%)–EDTA (0.2 g/l) into single cells for passaging at the ratio of 1 : 3. The culture medium was H-DMEM/F12 containing 100 U/ml of a penicillin-streptomycin mixture and 10% FBS.

### 2.3. Analysis of Cellular Phenotype of hUMSCs

In the logarithmic phase of growth, hUMSCs were digested with trypsin and rinsed with PBS, and then, the single cell suspension was aliquot into 10 tubes at 1 × 10^6^ cells/tube. Separately, mouse anti-human monoclonal antibodies (5 *μ*l/each) against CD11-PE, CD45-PE, CD73-PE, CD90-PE, CD105-PE, HLA-DR-PE, CD19-FITC, and CD34-FITC were added to the 8 tubes, while anti-mouse IgG1-PE and anti-mouse IgG1-FITC (each 7 *μ*l) were added to the other two tubes as isotype controls. After mixing the contents thoroughly, the tubes were incubated at 4°C for 30 min. Thereafter, the cells were rinsed with PBS and then centrifuged. The supernatant was discarded, and the cells were resuspended with 400 *μ*l of PBS for the followed analysis by flow cytometry.

### 2.4. Differentiation of hUMSCs into Neuron-like Cells

The hUMSCs were obtained at the 3rd passage of cells according to the logarithmic growth phase. The hUMSCs were separated into four groups including three groups treated with different dose of Edaravone (low-dose, LH; medium-dose, MD; and high-dose, HD) and one control group (Con). MSCs were placed in a cell culture chamber. When about 80% of the cells adhered to the wall, the whole culture solution was discarded. Neurobasal medium containing 50 ng/ml bFGF was first induced for 24 hours. After washing with PBS, LD, MD, and HD groups were, respectively, added with Edaravone concentrations of 0.65 mg/ml, 1.31 mg/ml, and 2.62 mg/ml for incubation with the Neurobasal medium. Then, the hUMSCs were induced in the incubator, and the morphological changes of the cells were observed by inverted phase contrast microscope.

### 2.5. Calculation of Positive Rate of Neuron-like Cells

Under the inverted microscope, 3 nonoverlapping fields were randomly selected; the total cell number and the number of neuron-like cells were counted, and the rate of neuron-like cells were calculated.

### 2.6. Immunochemistry Analysis

After incubation with Edaravone for 12 h, expression levels of neuronal-specific proteins NSE, nestin, and GFAP were detected by immunocytochemistry as follows. The cells were rinsed with PBS gently and fixed with 4% paraformaldehyde for 20 min at room temperature followed by the rinses with PBS three times for 5 min each time. The cell membranes were disrupted with 0.5% Triton-X-100 in PBS for 15 min at room temperature away from light and then incubated with 3% H_2_O_2_ at room temperature for 5 min. The cells were blocked by the normal goat serum at room temperature for 15 min. The blocked cells were induced by the primary antibodies against NSE, nestin, and GFAP at 4°C overnight. On the second day, the appropriate biotinylated secondary antibody was added for incubation at room temperature for 15 min, then followed by horseradish peroxidase-conjugated streptavidin at room temperature for 15 min. Finally, the cells were stained with freshly prepared DAB for 1 min and counterstained with hematoxylin before finally rinsed repeatedly with water. Under the inverted microscope, 3 nonoverlapping fields were randomly selected; the total cell number and the number of neuron-like cells were counted and the rate of neuron-like cells calculated.

### 2.7. RT-PCR Analysis

Total RNA was extracted by TRIpure Reagent (Aidlab Biotechnologies Co. Ltd., China) following the manufacturer's instructions and quantified with an Ultramicro ultraviolet visible light meter (Gene Company Limited, China). cDNA was synthesized from total RNA with EasyScript First-Strand cDNA synthesis supermix (TransGen Biotech, China) following the manufacturer's instructions. Semiquantitative PCR was performed using Taq PCR star mix (Genstar, China) with the following reaction conditions: 1 cycle of 94°C for 3 min; 30 cycles of 94°C for 30 s, T_m_ (°C) for 30 s, and 72°C for 30 s; and a final extension at 72°C for 10 min. A 10 *μ*l of each PCR product was analyzed by electrophoresis, and the gene expression levels were calculated using GAPDH as the internal control. The primers were designed using Primer 5.0 software and are shown: nestin: F-TCCAGAAACTCAAGCACCACT and R-TCCACCGTATCTTCCCACCT 342 bp, NSE: F-GGCACTCTACCAGGACTTTG and R-GCGATGACTCACCATAACCC 286 bp, GFAP: F-GTCCATGTGGAGCTTGAC and R-CATTGAGCAGGTCCTGGTAC 406 bp, and GAPDH: F-AGAAGGCTGGGGCTCATTTG and R-AGGGGCCATCCACAGTCTTC 258 bp.

### 2.8. Western Blot Analysis

Cell lysates were prepared with cell *RIPA* Lysis Buffer and phenylmethylsulfonyl fluoride, and protein concentrations were determined with an Ultramicro ultraviolet visible light meter (Gene Company limited, China).Total protein (16 *μ*g) of each lysate was electrophoresed in 10% SDS-PAGE gel and transferred to a polyvinylidene fluoride (PVDF) membrane (Solarbio, Beijing, China). The membranes were blocked by 10% nonfat milk for 2 h at room temperature and incubated with GFAP, nestin, and NSE primary antibodies at 4°C overnight. After rinsing with PBS for 10 min, the membranes were incubated with anti-rabbit IgG (1 : 2000, Affbiotech, USA) for 2 h at room temperature and visualized by Supersignal West Pico Chemiluminescent Substrate (Thermo Scientific, USA) using the Image alphaEaseFC system (Alpha Innotech, USA).

### 2.9. Continuous Culture of Neuron-like Cells

We chose a group of cells with the highest positive rate of nerve cell markers and stable expression. After 24 hours of induction, we clean it with PBS, replace the maintenance fluid, and continue training for five days. The maintenance fluid is formulated with Neurobasal medium containing 2% B27 and 50 ng/ml bFGF. The inverted phase contrast microscope dynamically observed the morphological changes of each group.

### 2.10. Real-Time PCR Analysis

Total RNA was extracted by TRIpure Reagent (Aidlab Biotechnologies Co. Ltd, China) following the manufacturer's instructions and quantified with Ultramicro ultraviolet visible light meter (Gene Company Limited, China). cDNA was synthesized from total RNA with EasyScript First-Strand cDNA synthesis supermix (TransGen Biotech, China) following the manufacturer's instructions. Real-Time PCR was performed using TB Green™ Premix Ex Taq™ II (Tli RNaseH Plus) by TaKaRa. The gene expression levels were calculated using GAPDH as the internal control. The primers were designed using Primer 5.0 software and are shown: SYN: F–CACTACTACAGAGGGAAAATGAATG and R-CACGGTGCCCAGACAGGT 108 bp, GAP43: F-GAAGGGGGAGGGTGATGC and R-CTTGGAGGACGGCGAGTTAT 130 bp, PSD95: F-GCGGAGCAACCCCAAAAG and R-GATGAACCCAATGTCGTCGG 196 bp, and GAPDH: F-ACGGATTTGGTCGTATTGGG and R-GATGAACCCAATGTCGTCGG 210 bp.

### 2.11. Statistical Analysis

All the data were expressed as mean ± s.e. SPSS19.0 was used for statistical analysis. One-way ANOVA was used in three or more group comparison followed by SNK method within two separate groups. *P* < 0.05 was considered to be significantly different.

## 3. Results

### 3.1. Growth and Morphological Changes of hUMSCs

**Figure 1:**
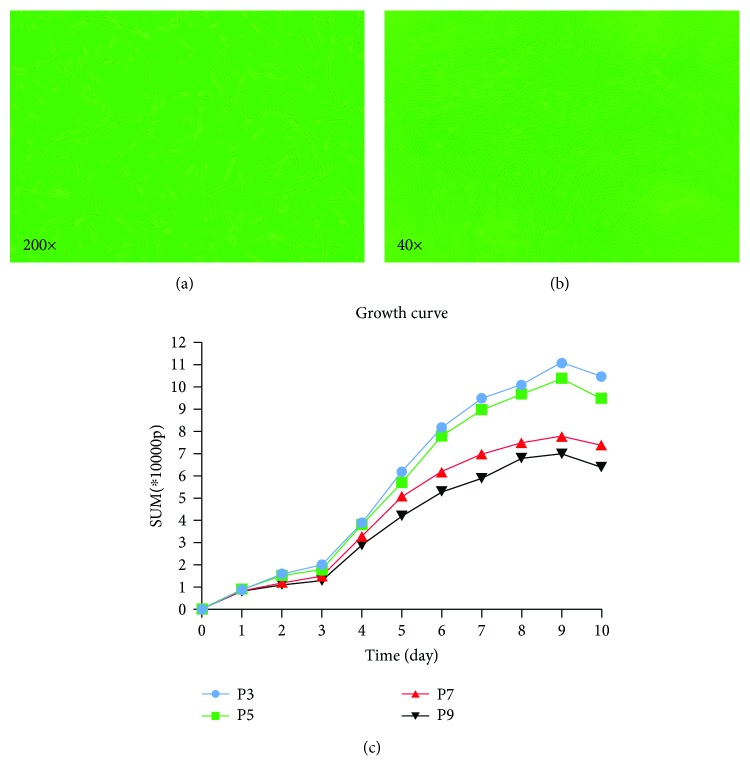
(a) (200x) Cells were elongated or long fusiform. (b) (40x) The cells were spirally divergent. (c) The growth curve of the 3rd, 5th, 7th, and 9th passage cells.

Primary cultured cells were passaged at the ratio of 1 : 1. After 12 hours of passage, most of the cells adhered to growth, and the cells were elongated or long fusiform (Figure 1(a)). With the increase of incubation time, the cells gradually became longer and adhered completely, and the morphology became flat. Cell growth was rapid, in about 5 days after passage can be completely adhere to the wall and grow. The cells had radial or spiral growth according to the ratio of 1 : 2 or 1 : 3 subculture. Continue to culture, the cells were overlapped showing the spiral divergent growth around the center, and the local density was increased (Figure 1(b)). The cells were cultured until the ninth passage, and the cell homogeneity and growth were good. Because of the relatively small number of the 1st and 2nd passage (P1 and P2) cells and the good growth condition of the 3rd passage (P3) cells, we selected the 3rd passage hUMSCs with good growth status. The growth curve is plotted in Figure 1(c).

### 3.2. Cellular Phenotype of hUMSCs

**Figure 2:**
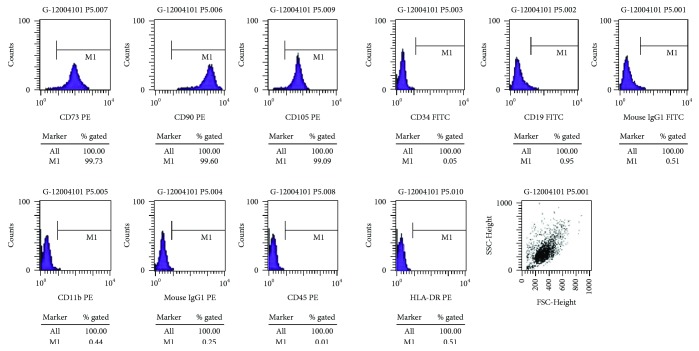
Cellular phenotype of hUMSCs detected by flow cytometry.

We detected the cell phenotype of the 2nd, 5th, and 10th passages of hUMSCs by flow cytometry and found that all generations of cells tested coexpressed CD105, CD90, and CD73 but not CD11b, CD34, CD19, CD45, and histocompatibility antigen HLA-DR (MHC-II) (Figure 2).

### 3.3. Morphological and Quantitative Changes of Neuron-like Cells in Each Group

**Figure 3:**
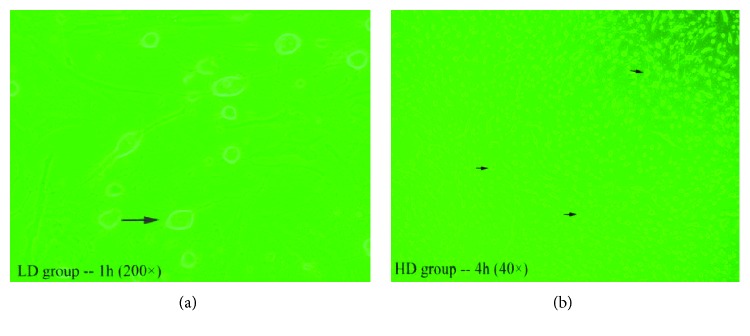
(a) (200x) Arrowheads show neuron-like cells. (b) (40x) A large number of neuron-like cells, arrowheads show neuron-like cells.

**Figure 4:**
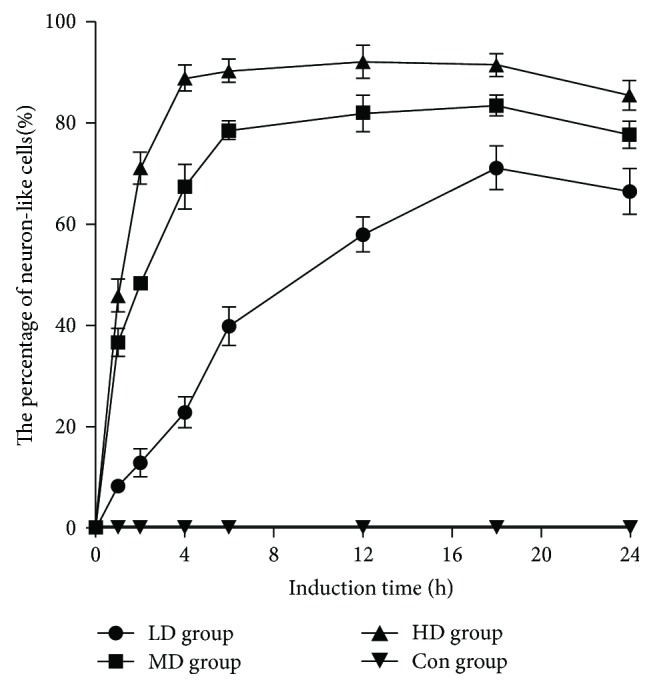
The percentage of neuron-like cells in each group was changed with the induction time.

The hUMSC incubation with Edaravone showed the morphological properties of neuron-like cells. After 1 h incubation with low dose of Edaravone, the hUMSCs were presented with the sporadic cell body contraction, the enhanced refraction, and the cell morphology which turned into a long oval or round and out of one or more elongated processes (Figure 3(a)). After 4 h, the number of protrusions increased, and the length of protrusions and the number of bifurcations increased. 12 h later, about 50% of the cells had the above changes; adjacent cells protruding protuberances will gradually intertwine connected into a network. The percentage of neuron-like cells reached the peak at 24 hours. The hUMSCs in the MD group were presented with obvious changes after adding Edaravone for 1 hour. In the MD group at 12 hours, the percentage of neuron-like cells reached the peak, which was much earlier than the LD group. In the HD group, a large number of cells became neuron-like cells at 1 hour; neuron-like cells reached a peak at 4 hours (Figure 3(b)) and remained at high rates of change until 24 hours. Blank group was the control group. There was no significant change in 24 h after continuous observation. Under the inverted microscope, 3 nonoverlapping visual fields were randomly selected; the total number of cells and the number of neuron-like cells were counted, and the rate of neuron-like cells was calculated. The result was expressed by mean ± s.e. The curve of the rate of neuron-like cells in each group is shown in Figure 4. There was significant difference in the positive rate between each group, *P* < 0.01. The cells in the HD group changed the most rapidly and maintained at a relatively high positive rate.

### 3.4. Result of Immunochemistry Analysis

**Figure 5:**
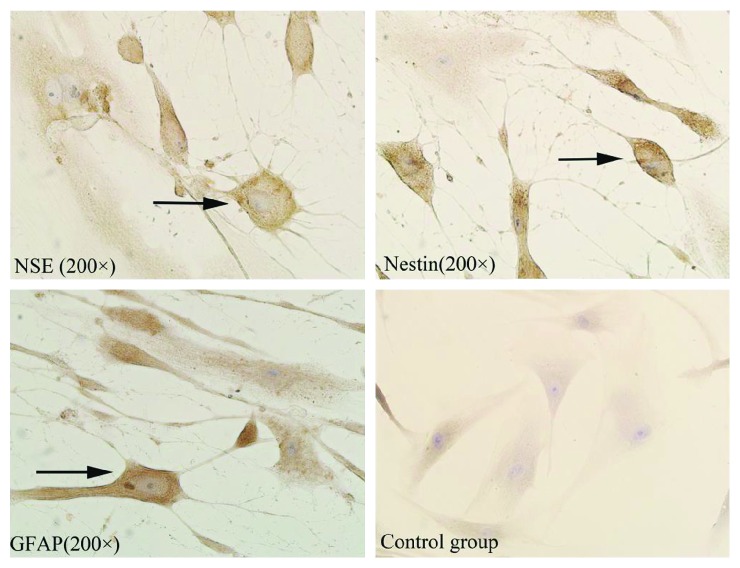
Immunohistochemical staining of neuron-like cells in treatment groups for NSE, nestin, and GFAP, respectively. The arrows in figures indicate neuron-like cells.

**Figure 6:**
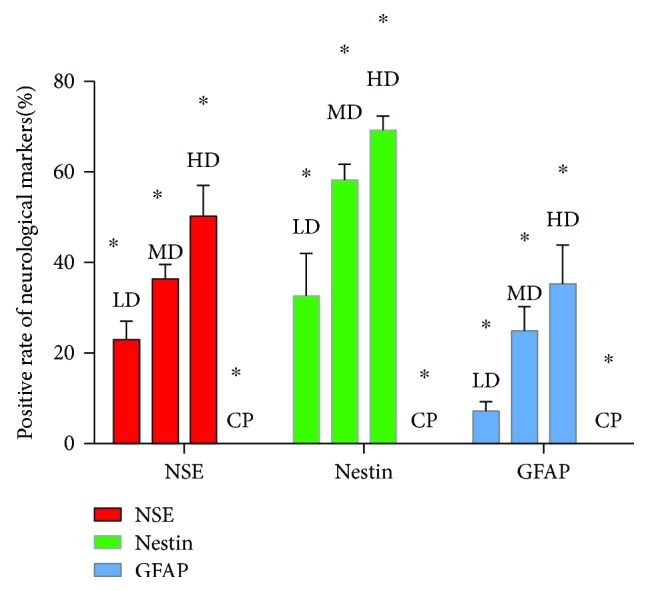
After 12 hours of induction, the positive rate of neuronal markers in each group.

The neurological markers were detected after different concentrations of Edaravone incubation for 12 hours. As shown in Figure 5, brown cell bodies and the purple nucleus were nestin, NSE, or GFAP-positive cells. By contrast, no cells positively expressed these markers in the control group. Under the inverted microscope, 3 nonoverlapping visual fields were randomly selected; the total number of cells and the number of neuron-like cells were counted, and the rate of neuron-like cells was calculated. After 12 hours of induction, the positive rate of neuronal markers in each group is shown in Figure 6. The result was expressed by mean ± s.e. Differences in expression levels of nestin, GFAP, and NSE between any two groups were significant (*P* < 0.05). The results showed that the expression of nerve markers in the HD group was the most.

### 3.5. RT-PCR Analysis Results of the HD Group

**Figure 7:**
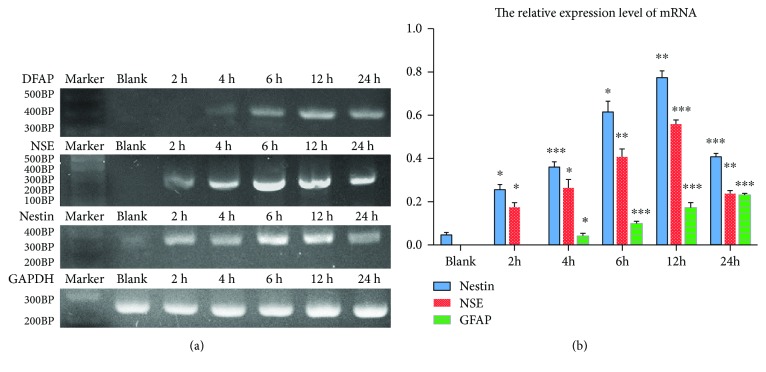
(a) Result of semiquantitative RT-PCR. (b) Relative expression level of mRNA.

The highest positive rate and stable expression of each nerve cell marker were detected in the HD group (2.62 mg/ml). Therefore, we use semiquantitative RT-PCR to detect the expression of mRNA in the HD group. According to the induction time, the groups were divided into blank, 2 h, 4 h, 6 h, 12 h, and 24 h. Through statistical neural marker expression amount and reference, to calculate the neural marker expression level, the result was expressed by mean ± s.e. RT-PCR results of NSE, nestin, GFAP, and GAPDH at each time point are shown in (Figure 7).

### 3.6. Western Blot Analysis Results of the HD Group

**Figure 8:**
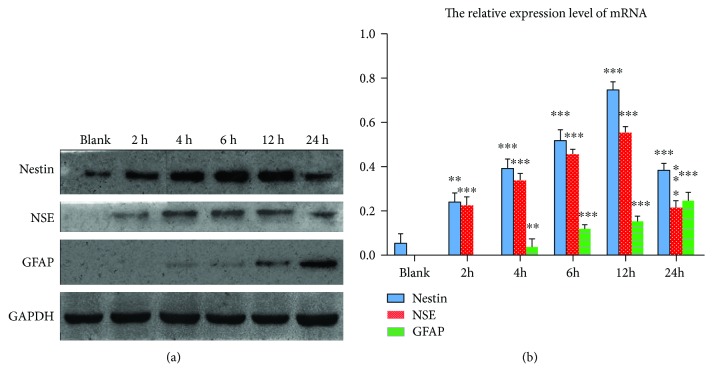
(a) Result of Western blot. (b) Relative expression level of protein.

We use Western blot analysis to detect the expression of protein in the HD group. According to the induction time, the groups were divided into blank, 2 h, 4 h, 6 h, 12 h, and 24 h. Through statistical neural marker expression amount and reference, to calculate the neural marker expression level, the result was expressed by mean ± s.e. Western blot results of NSE, nestin, GFAP, and GAPDH at each time point are shown in Figure 8.

### 3.7. Real-Time PCR Analysis Results of Specific Synapse Marker

**Figure 9:**
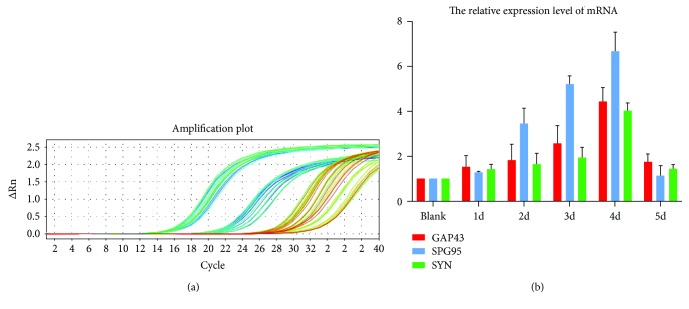
(a) Result of Real-Time PCR. (b) Relative expression level of mRNA.

We use Real-Time PCR to detect the expression of mRNA in the HD group. After replacement of maintenance fluid, nerve-like cells continued to be cultured for five days. According to maintenance culture time, the groups were divided into blank, 1 d, 2 d, 3 d, 4 d, and 5 d. Through statistical synaptic marker expression amount and reference, to calculate the synaptic markers expression level, the result was expressed by mean ± s.e. SYN, PSD95, and GAP43 at each time point are shown in Figure 9.

## 4. Discussion

With the rapid development of stem cell therapy research, stem cell transplantation is considered to be a potential treatment. At the same time, mesenchymal stem cells as a new source of cell therapy are of concern to a large number of scholars and researchers [17, 18]. In theory, pluripotent embryonic stem cells and the inner layer of blastocysts are the ideal cell transplantation option. However, the application of these cells is ethically restricted and has teratogenicity and tumorigenicity. MSC can have in vitro long-term survival and constantly amplify and induce differentiation into nerve, vascular endothelium, myocardial, corneal endothelium, cartilage, islet, and other functional cells [5]. The nervous system has little ability to repair itself after being damaged, so the nerve cells that are targeted by neural tissue engineering may be able to repair the damaged nerve tissue. Previous studies have shown that bone marrow MSCs, neural stem cells, adipose MSC, umbilical cord MSC, umbilical cord blood MSC, and placental stem cells from different species and sources can be used for the treatment of neurological diseases. Shen et al. [19] report the treatment of ischemic or some degenerative disease caused by nervous system dysfunction; mesenchymal stem cells can provide a stable source of nutrition and reflect its multidirectional differentiation potential. At the same time, MSC do not appear to decrease with the number of passages, and there is no risk of differentiation with time [20]. This conclusion has been demonstrated in models of intracerebral hemorrhage and Parkinson's disease in rats and spinal cord injury in primates [21, 22]. Previous adult stem cell research mostly focused on bone marrow-derived mesenchymal stem cells. There are limitations and defects in bone marrow MSC because of limited number and difficulty in obtaining. The number and differentiation potential of bone marrow mesenchymal stem cells will decrease with age. At present, human umbilical cord mesenchymal stem cells (hUMSCs) are popular cells in cell culture. hUMSCs have the advantages of strong proliferative ability, low immunogenicity, stable amplification in vitro, wide source, without any ethical restriction, and can be divided into multiple germ layers. At present, there is no study on the differentiation of human umbilical cord mesenchymal stem cells induced by Edaravone. Therefore, we used Edaravone to induce human umbilical cord mesenchymal stem cells. More experiments show that under certain conditions of culture, hUMSCs spontaneously tend to differentiate into neuron-like cells and have a good protective effect of neurons [23]. At this stage, the use of cell therapy in the treatment of neurological diseases is divided into the following two methods [24, 25]. One is the purpose of the cells through a variety of ways into the body, relying on its own differentiation potential, and the impact of the environment plays a therapeutic effect. In another method, cells are first differentiated into cells of interest by various means and then into the body to play a therapeutic effect.

The most reported method of induction into nerve cells is divided into three kinds: cell nutrition factor, antioxidant, and nerve cell coculture method. In general, the main factors of cell nutrition are bFGF, transforming growth factor-*β*, and BDNF. Antioxidants are mainly DMSO, compound or single herb, or active ingredients of traditional Chinese medicine. The neural cell coculture method is to coculture MSCs with neural cells. There are also reports that microRNA-124 can also regulate the directional differentiation of MSCs to neurons [26]. These neuron-like cells can express a variety of neurological markers, such as nesin, GFAP, NSE, NF-H, and MAP-2. In this study, different concentrations of Edaravone were used to induce hUMSCs. It was found that the astigmatism of hUMSCs became stronger, and the cells protruded elongated protrusions, which had the morphological characteristics of neuron-like cells. The results of immunocytochemistry showed that NSE, GFAP, and nestin were positive, and the induction efficiency of 2.62 mg/ml group was significant. The results of RT-PCR and Western blot showed that the expressions of NSE, GFAP, and nestin in the induced group were significantly higher than those in the blank control group. And 2.62 mg/ml is the higher concentration available, close to its maximum solubility in L-DMEM [27]. This study demonstrates that Edaravone can effectively induce hUMSCs to differentiate into neuron-like cells and the concentration of 2.62 mg/ml for the appropriate induction concentration.

The mechanism of each induction method is also different, such as bFGF induction mechanism as a neurotrophic factor in promoting cell mitosis in the process of simulation of embryonic neural cell growth microenvironment, thus leading to hUMSC differentiation into nerve cells. The induction mechanism of coculture may be related to the secretion of various nerve growth factors from various nerve cells. In this growing microenvironment, hUMSCs tend to differentiate into neuron-like cells, also providing the conditions for axonal development [28, 29]. Tetramethylpyrazine can promote the differentiation of hUMSCs into neuron-like cells by inhibiting the expression of Ca2+ signaling, promoting the increase of cAMP content in the second messenger and changing the expression of MEK-ERK signaling pathway [30]. Chemical antioxidant *β*-mercaptoethanol is also regulating the second messenger cAMP content, thus initiating the PKA pathway to promote MSC differentiation [31]. In recent years, with the development of biological tissue engineering technology, some scholars have proposed that using certain cell scaffold compounds can promote the differentiation of MSCs [32]. Edaravone is widely used in the treatment of ischemic stroke and a variety of spinal cord injury. Edaravone has the effect of reducing cerebral edema, improving cerebral ischemic symptoms, protecting brain tissue, and so on. It is a kind of oxygen free radical scavenger with good curative effect. Edaravone has antioxidant, anti-ischemia and reperfusion injury, antifibrosis, inhibition of nerve cell damage, and other effects; it can inhibit the peroxidation to protect the brain cells [33]. Edaravone is a kind of brain protective agent, which can reduce the death of nerve cells. In brain hemorrhage and cerebral infarction experiments, it can inhibit the decrease of local cerebral blood flow around the infarct. We hope to use cell transplantation to improve the neuron injury caused by brain injury, bleeding, infarction, and the adverse effects of Parkinson's disease, amyotrophic lateral sclerosis, and other degenerative diseases. We look forward that cells derived from Edaravone can also play a role in brain protection, providing cell support for the next further experiments. Therefore, in this study, we used Edaravone to induce hUMSCs to differentiate into neuron-like cells. At the same time, it was found that those neuron-like cells induced by Edaravone had the electrophysiological basis for neuronal cells [34]. However, the induction mechanism of Edaravone is still unclear at this stage. Weissmann et al. [35] and other studies have shown that in the growth and development of nerve cells, the cytoskeleton played a key role. Doherty [36] and other studies have pointed out that in the development of nerve cells, Shh signaling pathway has an important role; it can be conducive to the survival of nerve cells in the microenvironment. The next research direction is whether the Shh signaling pathway and cytoskeleton play an important role in the induction of hUMSCs into neuron-like cells by Edaravone.

Synaptic plasticity has long been recognized as the basis of neurocognitive biology. Many studies have found that memory can be preserved for a long time by the brain at the synapse level [37]. The existing proteins are modified to give signals to the nucleus. So a specific gene is expressed. Gene products are transported to synapses, and new proteins are synthesized locally, thus establishing new synapses and forming new information transfer functions [38]. This process is called synaptic plasticity. Changes in synaptic markers can reflect changes in morphological plasticity. Synaptophysin (SYN), as one of the most important markers of synaptogenesis, plays an important role in the process of synaptogenesis and differentiation [39]. Postsynaptic density 95 is mainly involved in maintaining synaptic structure and plays an important role in the regulation of synapse morphology [40]. Growth-associated protein 43 (GAP43), as a specific phosphorylated protein, is mainly located in the presynaptic membrane of neurons. It is widely involved in the development, differentiation, and regeneration of the nervous system and is closely related to the formation and plasticity of synapses [41]. Therefore, we examined the expression levels of SYN, GAP43, and postsynaptic density 95. However, whether the obtained neuron-like cells have synapses capable of transmitting information needs further neuro-electrophysiological studies.

At the same time, hUMSCs cannot only directly inhibit the growth of some tumors in vivo and in vitro but also easily accept foreign gene modification and have the characteristics of target migration to tumor tissue, damaged tissue, and chronic inflammatory response sites. We would like to discuss whether the cells can also have the characteristics of nerve cell protection and tumor cell apoptosis by using eukaryotic cell transfection. There are also many difficulties in introducing nerve cells which are differentiated into cells into the body and have a therapeutic effect. In the next days, we still need to continue efforts.

## Data Availability

The data used to support the findings of this study are included within the article.
